# Impact of COVID-2019 on school attendance problems

**DOI:** 10.7189/jogh.11.03084

**Published:** 2021-07-31

**Authors:** Gina Nathwani, Adeel Shoaib, Alliya Shafi, Toshi A Furukawa, Nguyen Tien Huy

**Affiliations:** 1 American University of Caribbean School of Medicine, Cupecoy, Sint Maarten; 2 Department of Health Promotion and Human Behavior, Kyoto University Graduate School of Medicine, School of Public Health, Kyoto, Japan; 3 School of Tropical Medicine and Global Health, Nagasaki University, Japan

Disputed education and school attendance problems (SAPs) during the Coronavirus Disease 2019 (COVID-19) pandemic may tremendously impact child health and education. Limited studies evaluate the socioeconomic and personal factors behind SAPs associated with the COVID-19 pandemic. School refusal, truancy, school withdrawal and school exclusion are considered SAPs [1]. Prior to the COVID-19 pandemic, self-reported school absences in a population of 4344 Belgian students found that of those with one unauthorized absence from school: 49.4% were categorized as truant, 17.4% as school refusal and 33.2% as school withdrawal [2]. There are four types of SAPs: school refusal, truancy, school withdrawal and school exclusion. Currently, some studies are being conducted that analyze transmission and attendance rates in children returning to school, in-person or online, after periods of abrupt closure [3,4]. Additionally, school refusal can be attributed to parental fear of their children being susceptible to COVID-19 associated multisystem inflammatory syndrome [5]. Currently, no studies exist that directly address the effect of COVID-19 on SAPs, academic performance, or child mental health and functioning. We aim to review and categorize these four SAPs, associated factors of decreased student attendance, and the impact of this disruption amidst the COVID-19 pandemic. We hypothesize that COVID-19 related SAPs will increase compared to pre-pandemic levels as mental health issues rise and virtual classes hinder academic performance.

## SCHOOL REFUSAL

School refusal, as defined by Berg and colleagues (1997), is a type of SAP characterized by a young person’s reluctance or refusal to attend school which leads to prolonged absences and remaining at home during school hours [6]. According to Heyne et al. (2006), school refusal is defined as a young person conveying emotional conflict at the idea of attending school [7]. In school refusal the young person makes no attempt to hide their absence from their parents, and school attendance is associated with emotional distress [1]. This often manifests into excessive fearfulness and the reluctance to attend school. As such, a high prevalence of mood and disruptive behaviors occur in youth that refuse to attend school [8]. A recent United States (US) study during the COVID-19 pandemic found student school refusal anxiety and preference for remote learning associated with pre-existing health conditions within families, as well as with Black or Asian ethnicity [9]. In a recent survey of 730 US parents of school-aged children, school refusal was associated with parental fear of their child’s susceptibility to COVID-19 multi-systemic inflammatory syndrome [5].

**Figure:**
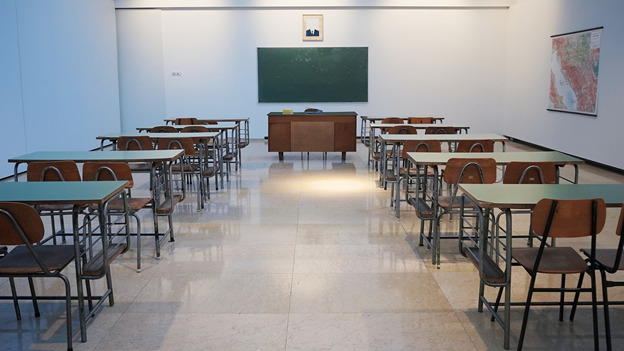
Photo: From the exhibition The Nineties: A Glossary of Migrations.

## TRUANCY

Heyne et al. (2018) describe truancy as a young person’s absence from school or class for the whole day or most of the day [1]. This absence occurs without the permission of the school authorities and an attempt to conceal this from their parents is made [1]. The overall prevalence of US student truancy is estimated at 11% between 2002 to 2014 even with reduction efforts [10]. Truancy is highest among Hispanics and African American compared to their non-Hispanic white counterparts. Truants are more likely to be older and report substance use such as alcohol, marijuana, and tobacco compared to their non-truant counterparts [10]. Truant youth are not a homogenous group; some are low risk and doing well academically in school, while others are of higher risk with poor academic performance and engage in substance use and delinquency [11]. Currently, no studies exist on truancy during the COVID-19 pandemic.

## SCHOOL WITHDRAWAL

School withdrawal is defined by parents withdrawing a child from school deliberately due to their own needs [1]. The following is an encompassing definition of school withdrawal: “school withdrawal occurs when a young person’s absence from school, due to late arrivals, missing whole school days, weeks, months or years is: 1. not concealed from the parent(s) and 2. attributable to parental effort to keep the young person at home or little parental effort in getting the young person to school” [1]. School withdrawal can be intentional or unintentional and is also further characterized by family-based or school-based reasons [12]. Examples of family-based reasons as determined by Kearney and colleagues (2004) include children helping with a parent’s paid work, reducing parental separation anxiety, or a form of punishment [12]. School-based reasons include hiding information (mental disorder, incomplete homework, or maltreatment) from school staff, pursuing homeschooling uselessly, or letting a child stay home if another child has a day off from school [13]. Currently, no studies exist on school withdrawal during the COVID-19 pandemic.

## SCHOOL EXCLUSION

School exclusion occurs due to school-based decisions, such as the use of inappropriate disciplinary measures, not receiving the proper resources (eg, special needs), or not being able to meet school-based performance requirements [1]. This SAP is the result of decisions imposed on parents and children by their school or academic institution. School exclusion is likely more prevalent in children diagnosed with psychiatric disorders [14]. Pre-pandemic studies found children of immigrants may face school exclusion if the child experiences systemic discrimination [15]. A recent study focused on COVID-19 related school closure suggested that partial school re-openings may benefit the youngest children that cannot benefit from online learning, and decrease rates of potential school exclusion later in their education [16].

## SAPS AND FUTURE RECOMMENDATIONS

Currently, a paucity of research exists highlighting the relationship between COVID-19 school reopenings, associated SAPs, particularly truancy and school withdrawal, and the impact on children. Studies conducted on the rate of COVID-19 transmission and case numbers associated with return to in-person schooling are mostly inconclusive. However, a recent study concluded that COVID-19 is more likely transmitted from adults compared to children, and that student transmission rates after return to school should be lower than expected [17]. A cross-sectional survey study of US parents prior to school re-openings concluded that school attendance would primarily be affected by socioeconomic factors [5]. The inability to maintain appropriate social distancing and preventative precautions may hinder students from lower income areas that attend underfunded and overcrowded schools from attending class in person [5]. We suggest development of policies that ensure children of lower socioeconomic status receive a safe education which would be paramount in SAP prevention. A recent UK study showed an increase in the incidence of mental health problems from 10.8% in 2017 to 16% in July 2020 in children aged 5-16 [18]. A recent pre-pandemic study found lower academic efficacy, subpar academic performance, symptoms of depression and anxiety, and a lack of self-esteem in youth with SAPs [19,20]. A US study found a statistically significant increase in positive suicide risk screens and suicidal ideation among youths 11-21 years old that visited a pediatric emergency department during the COVID-19 pandemic compared to pre-pandemic screening [21]. Additionally, truants are more likely to suffer from forms of depression and suicidal ideation [22,23]. We suggest increased mental health support and parental awareness of their child’s well-being related to SAPs, particularly during the pandemic.

In addition to policies regarding SAPs, parental decisions will play a critical role for student attendance during the COVID-19 pandemic and the determination of whether a student will be labeled a truant [11]. As most academic institutions opt for remote learning in addition to in-person learning, policies must determine the consequences of truancy and cases of absences during the COVID-19 pandemic. We suggest that future research focus on COVID-19 related SAPs such as school refusal, truancy, school withdrawal and school exclusion, and the impact on child physical and mental health. New academic guidelines implemented to decrease risk of viral transmission, and parental and child beliefs may significantly impact SAPs. Academic, parental and child necessities, priorities, and beliefs may require compromise to ensure children receive quality schooling and prevention of SAPs. With this knowledge, policy makers, physicians, teachers and parents may work together to mitigate SAPs during the upcoming transition into in-person learning, and in possible future pandemics.

Future questions to be explored include how different school districts define school attendance during the COVID-19 pandemic and how their curriculum will be adapted to prevent absenteeism through retrospective surveys. Additional questions that need to be addressed include how school policies regarding attendance will adapt as the COVID-19 vaccine becomes more readily available and whether the vaccine will be mandated to return to in-person classes. Mixed method studies including cross-sectional surveys and short interviews can be conducted to gage the parent’s plan to return their children to in-person learning for the Fall 2021 school year as the vaccine becomes more accessible. It would also be crucial to explore whether other factors, including the child’s willingness to return, socioeconomic factors, and vaccine uptake will play a role in this decision.

## CONCLUSION

The current COVID-19 pandemic resulted in the abrupt closure of schools disrupting in-person education internationally. There are currently no studies directly addressing the effect of COVID-19 and SAPs on academic performance, or child mental health and functioning. However, children with SAPs seem to be more at risk of mental health issues, which may increase during the pandemic. Special attention by care-providers and academic institutions should be provided to decrease occurrence of SAPs and associated factors.
